# Prediction of Disorientation by Accelerometric and Gait Features in Young and Older Adults Navigating in a Virtually Enriched Environment

**DOI:** 10.3389/fpsyg.2022.882446

**Published:** 2022-04-25

**Authors:** Stefan J. Teipel, Chimezie O. Amaefule, Stefan Lüdtke, Doreen Görß, Sofia Faraza, Sven Bruhn, Thomas Kirste

**Affiliations:** ^1^Deutsches Zentrum für Neurodegenerative Erkrankungen (DZNE) Rostock/Greifswald, Rostock, Germany; ^2^Department of Psychosomatic Medicine, University Medicine Rostock, Rostock, Germany; ^3^Mobile Multimedia Information Systems, Institute for Visual and Analytic Computing, University of Rostock, Rostock, Germany; ^4^Institute for Enterprise Systems, University of Mannheim, Mannheim, Germany; ^5^Institute for Sports Science, University of Rostock, Rostock, Germany

**Keywords:** navigation, virtual reality, aging, visuo-spatial abilities, executive function, gait, actimetry

## Abstract

**Objective:**

To determine whether gait and accelerometric features can predict disorientation events in young and older adults.

**Methods:**

Cognitively healthy younger (18–40 years, *n* = 25) and older (60–85 years, *n* = 28) participants navigated on a treadmill through a virtual representation of the city of Rostock featured within the Gait Real-Time Analysis Interactive Lab (GRAIL) system. We conducted Bayesian Poisson regression to determine the association of navigation performance with domain-specific cognitive functions. We determined associations of gait and accelerometric features with disorientation events in real-time data using Bayesian generalized mixed effect models. The accuracy of gait and accelerometric features to predict disorientation events was determined using cross-validated support vector machines (SVM) and Hidden Markov models (HMM).

**Results:**

Bayesian analysis revealed strong evidence for the effect of gait and accelerometric features on disorientation. The evidence supported a relationship between executive functions but not visuospatial abilities and perspective taking with navigation performance. Despite these effects, the cross-validated percentage of correctly assigned instances of disorientation was only 72% in the SVM and 63% in the HMM analysis using gait and accelerometric features as predictors.

**Conclusion:**

Disorientation is reflected in spatiotemporal gait features and the accelerometric signal as a potentially more easily accessible surrogate for gait features. At the same time, such measurements probably need to be enriched with other parameters to be sufficiently accurate for individual prediction of disorientation events.

## Introduction

Aging is associated with a decline in walking ability (Baudendistel et al., 2021) and cognitive performance (Iachini et al., 2009). These changes become particularly evident in dual-task conditions. For example, older people have difficulties walking and navigating in a new environment (Lithfous et al., 2013; Lester et al., 2017), resulting in reduced wayfinding abilities. These changes are even more pronounced during the transition from healthy aging to cognitive impairment and dementia (Gazova et al., 2012; Cohen and Verghese, 2019; Costa et al., 2020). They represent a high burden on older people and lead to fear of getting lost, social withdrawal, and a subsequent decrease in physical mobility (Panel on Prevention of Falls in Older Persons, American Geriatrics Society and British Geriatrics Society, 2011).

At the same time, wayfinding problems are amenable to technical assistance. Navigation systems are already part of our everyday environment; they support drivers and pedestrians, for example. For older people and people with cognitive impairments, in particular, it is important that assistance systems do not replace remaining cognitive abilities, but rather make use of them. Previous work has shown that habitual use of navigation aids may decrease spatial memory performance even in cognitively healthy people (Dahmani and Bohbot, 2020). Current technology development is therefore aimed at situation-aware navigation assistance that supports the user only when necessary (Teipel et al., 2016). Such systems require accurate detection of navigation behavior, especially real-time detection of episodes of disorientation before the user is lost (Yordanova et al., 2017).

Previous studies used experiments in virtual reality (VR) environments to assess spatial orientation (Zakzanis et al., 2009; Kizony et al., 2017; Tascon et al., 2018; Costa et al., 2020; Paliokas et al., 2020). VR approaches are highly controlled but lack the dual-task characteristic of combining spatial navigation with walking. One previous study found that navigational performance results were comparable between a VR and a real-world navigational test in young and older cognitively normal adults and people with dementia (Cushman et al., 2008), but VR testing alone obviously does not allow assessment of gait and motion features during spatial navigation. On the other hand, several studies used wearable sensors to assess the gait and movement characteristics of cognitively normal older people and people with dementia in real-life situations (Becu et al., 2020; Mc Ardle et al., 2021; Pawlaczyk et al., 2021; Weizman et al., 2021). Some of these real-world studies were primarily aimed at exploring different components of spatial orientation in normal human behavior and the underlying neural basis but did not aim to map the full range of navigational behavior in everyday situations (Wei et al., 2020). Other studies mainly focused on the early detection of dementia symptoms using gait characteristics in real-world environments (Mc Ardle et al., 2021; Mulas et al., 2021; Weizman et al., 2021) or under dual task conditions (Oh, 2021).

In a previous study, we had assessed whether accelerometric features from wearable sensor devices were useful to identify episodes of disorientation even before an individual has deviated from the intended route (Schaat et al., 2019). We found that accelerometry-detected episodes of disorientation with an area under the receiver operating characteristics (ROC) curve (AUC) of 75% and 79% correctly allocated disorientation episodes in people with mild cognitive impairment (MCI) or dementia moving through an urban environment (Schaat et al., 2019). This level of accuracy suggested that there were relevant features in the accelerometric signal to detect disorientation at the group level. At the same time, the accuracy was not high enough for individual situation detection. In addition, people with dementia or MCI experienced a relatively small number of disorientation episodes, which limited the training of an accurate model based on positive events (Schaat et al., 2019).

Here, we transferred our previous approach to the better-controlled environment within the Gait Real-Time Analysis Interactive Lab (GRAIL) system. The GRAIL consists of a physical treadmill combined with a large hemisphere screen (Amaefule et al., 2020). In our experiment, the GRAIL screen featured a virtual representation of the city center of Rostock, resembling the environment of the previous real-world experiment (Schaat et al., 2019). Participants were asked to navigate through this environment while walking on the treadmill. In a previous pilot study, we showed that this set-up was feasible for use with older participants, including people with cognitive decline, and allowed us to record a comprehensive set of predictive features, including accelerometry, gait features, and physiological signals (Amaefule et al., 2020). In addition, we were able to induce disorientation episodes by removing landmarks from the virtual environment to provide more instances for model training. The key role of landmarks for spatial orientation in virtual environments has previously been shown (Caffo et al., 2018). In this study, we presented the results of this approach in young and older adults without manifest cognitive impairment. As a primary aim, we wanted to determine whether a combination of accelerometry and gait characteristics was accurate enough to immediately detect episodes of disorientation. We hypothesized that the accerelometric and gait features may yield sufficient accuracy for individual detection of disorientation episodes in real time. Especially, we expected a level of accuracy above 80% for the binary outcome of oriented *vs*. disoriented. As a secondary aim, we determined whether the number of disorientation events per participant was associated with cognitive scores and aggregated accelerometric and gait characteristics. The results of this study will be relevant to the design of experiments with individuals with manifest cognitive decline and also to the design of future real-world experiments targeting situation-aware navigation aids.

## Materials and Methods

### Subjects

For the ongoing GRAIL study, we recruited three groups of participants: mobile, physically and cognitively healthy younger (18–40 years) and older (60–85 years) participants, and physically healthy persons with diagnosed MCI or mild dementia due to AD (Age: 60–85 years, MMSE: 15–27) according to NIA-AA criteria (Albert et al., 2011; McKhann et al., 2011).

Patients and healthy older adults were recruited from the memory clinic of the Rostock University Medical Center, while the healthy young adults were recruited from within the University of Rostock student community. Exclusion criteria for all groups were other neurological conditions besides MCI or dementia in the patient group, inability to understand task instructions and questionnaire items, deaf-muteness, and blindness.

Due to the COVID pandemic restrictions, recruitment of patients with MCI and dementia was not possible for a longer time interval so only four patients had been recruited during the planned run-time of the project. Therefore, for the current analysis, we used only the data of a subset of 28 older and 25 young cognitively healthy participants that had complete data sets and behavioral annotation.

This study has been reviewed and approved by the Ethics committee of the Rostock University Medical Center (Approval No. A 2019-0062).

### Experimental Set-Up

The experimental set-up has been described before (Amaefule et al., 2020). In brief, the participants were guided along a path in the virtual environment. Afterward, they were set back to the starting point and asked to walk the same path again, this time unguided. Navigation was possible by walking more to the left or right on the treadmill; this rotated the participant’s position in the virtual environment to the left or right. The navigation route consisted of 14 major decision points (DP) which were primarily locations at which the participant had to decide to either continue in a particular direction, make a turn, or identify the goal position. For half of the healthy young or older subjects (the experimental group), phases of disorientation were induced by changing landmarks or decision points in the VR environment. These changes included (a) moving a landmark from one intersection to the next intersection, (b) adding a decision point, that is, an intersection, (c) blocking a road, and (d) moving the goal indicator to a different location. Overall, five locations were manipulated in the experimental group as follows: DP4 – a red pillar was moved from DP7 to DP4; DP9 – the road was blocked; DP11 – a new path was introduced; DP13 – the color of the pillar was changed to red; DP14 – the goal location was moved a little further away to DP14a. No changes to the environment were conducted in the control group.

Before the experiment, the participants were familiarized with the depicted city center by briefly showing them a map, such that problems in wayfinding would be due to disorientation instead of exploration in an unknown environment. We recorded spatiotemporal and kinematic gait parameters through the GRAIL system. In addition, we recorded accelerometric signals from three wearable sensors on the left wrist, right ankle, and chest, respectively, that each contained a three-axes accelerometer and three-axes gyroscope sampled with 64 Hz. Additionally, the chest sensor recorded an electrocardiogram (ECG, 1,024 Hz), and the wrist sensor recorded electrodermal activity (EDA, 32 Hz).

The experiments were video-recorded for subsequent offline annotation of behavior.

Randomization of the young and older participants into the experimental or control group was carried out using the program Research Randomizer, accessible at https://www.randomizer.org.

### Behavior Annotation

An offline annotation procedure was applied to the video data recorded during the orientation task, for assessing the observable orientation behavior of the participants using the ELAN 5.8 tool (Wittenburg et al., 2006). As a coding scheme, we used an adequate adaption of the coding scheme provided by Yordanova et al. (2017). The same scheme had been used in one field study before (Schaat et al., 2019). This coding scheme also covers aspects of orientation behavior, which were beyond the scope of wayfinding in our VR set-up (e.g., behaviors associated with attention to traffic). For this reason, we adapted the coding scheme to capture only those behaviors that are obtainable within our virtual reality set-up.

Specifically, to identify instances of disorientation, we annotated when participants showed wandering behavior (i.e., non–goal-directed walk), communication behavior (i.e., asking for help when disoriented), topological orientation (i.e., trying to orient themselves based on the surrounding environment), or spatial orientation (i.e., trying to orient themselves based on landmarks). In addition, different types of errors that are associated with disoriented behavior were annotated (i.e., initiation, realization, sequence, and completion errors). The annotations were being evaluated based on the level of agreement between two annotators independently rating the data of five individuals, resulting in a Cohen’s kappa of 0.87.

For the current analysis, the different types of disorientation behaviors were collapsed into a single feature of disorientation to provide a binary outcome of oriented *vs*. non-oriented state at a given time interval.

### Neuropsychological Assessment

Neuropsychological assessment was only conducted on the older participants and the MCI or dementia patients. The assessment included the CERAD neuropsychological battery (Morris et al., 1989), the Rey–Osterrieth Complex Figure Test (Rey, 1941; Osterrieth, 1944), and the Perspective Taking/Spatial Orientation Test (PTSOT) (Hegarty and Waller, 2004). Cognitive domain composite scores assessing visual memory, executive functions, visuospatial constructional ability, and spatial orientation were computed by transforming raw scores of single tests to z-scores (Coley et al., 2016; Voss et al., 2018). Each of these domain scores were calculated as the mean score of specific tests, after transformation to z-scores. The visual memory composite included the delayed figural recall scores from the CERAD and the Rey Complex Figure Test after 3 min; the visuospatial composite included the direct figure copy scores from CERAD and the Rey Complex Figure Test. For executive function, we used the ratio of Trail Making Test B to A, and for the domain of spatial orientation, we included cognitive scores from the Perspective Taking/Spatial Orientation Test.

### Predictors

We included spatiotemporal and kinematic gait parameters from the GRAIL system, as well as the mean accelerometric signal from ankle, chest, and wrist-worn sensors and variability of these measures. To reduce the dimensionality of the models for the association analysis, we selected *a priori* features of interest. These included the ankle, wrist, and chest-worn mean accelerometric signal as well as the mean values of the spatiotemporal gait characteristics of walking speed, step length, stride time, step width, stance time, and swing time (Beauchet et al., 2017). The explorative multivariate models for real-time detection were allowed to select across all spatiotemporal and kinematic gait features (Lohman et al., 2011) first and second moments (mean and variance), the accelerometric signal means and variances at the time point of behavior assessment as well as the time-lagged features one, two, or three time intervals before the rated behavior (lagged features). Supplementary Table 1 provides an overview of the feature sets defined for the different analyses.

### Gait and Accelerometric Data Preprocessing

Accelerometric data, gait parameters, as well as video annotations were synchronized by an event-based mechanism (participants performed a distinctive movement at the beginning of the recording, which could be easily located in all sensors). The data were resampled at 100 Hz using cubic spline interpolation. We then aggregated the data in non-overlapping segments of length 10 s. Specifically, for the accelerometric data, we computed the mean, variance, skewness, and kurtosis of the magnitude of each of the three sensor positions, resulting in 12 features per segment. For the spatio-temporal gait parameters (walking speed, step length, stride time, stance time, swing time, and step width), the mean and coefficient of variation (CV) were computed for each segment. The CV was calculated for each gait parameter as the ratio of the standard deviation to the mean multiplied by 100.

We assigned a binary disorientation label to each 10-s segment based on the video annotation using the following rule: Whenever a navigation error or disoriented behavior was noted at any time during the segment, the segment was labeled as “disoriented.” Conversely, if neither a navigation error nor a disoriented behavior was noted during the segment, the segment was labeled “not disoriented.”

### Statistical Analysis

Unless otherwise noted, all statistical analyses were performed using R statistical software, version 4.1.2, accessed *via* R Studio version 2021.09. Analyses were conducted in a Bayesian framework to allow estimation of model plausibility and determining effect sizes with credibility intervals. Demographic characteristics were compared between experimental groups using the Bayesian *t*-test or the Chi-square test as appropriate using Jeffreys’s Amazing Statistics Program (JASP) 0.16 with default priors.

Subsequently, we conducted two groups of analyses:

The *first group of analyses* (A1) used the disorientation data aggregated across the entire observation period per participant. We selected two readouts for disorientation: the number of disorientation per subject during the navigation experiment (henceforth called disorientation counts) and the percentage of the length of the disorientation episodes relative to the overall length of the experiment per subject (henceforth called disorientation percentage).

First, we determined the regression of aggregated disorientation data on cognitive scores (only in old people) and aggregated accelerometric and gait features (in young and old people). We used generalized linear models with disorientation counts and percentage, respectively, as dependent variables, and cognitive scores and aggregated accelerometric and gait features as independent variables, respectively, controlling for age, gender, and experimental condition. The dependent variable (count data) was not normally distributed, therefore we fitted a Poisson regression model using the R library “brms.” We compared the fit of the Poisson with the Gaussian regression model using leave-one-out cross-validation for Bayesian models with the R library “loo.”

The *second group of analyses* (A2) used the binary variable of oriented (0) *vs*. disoriented (1) during each of the 10-s intervals as the dependent variable in all individuals. To enrich for disorientation episodes, we only considered time intervals during decision points (see Supplementary Table 2 for the proportion of disorientation events per decision point).

First (A2.1), we used the Bayesian mixed-effects logistic regression models with accelerometric or gait features at each of the 10-s intervals as independent variables, controlling for age, gender, and experimental condition as fixed effects covariates, and with a random intercept for patients as random effect variable (observations nested within patients). These models were calculated using the R library “brms.

Second (A2.2), we determined, whether single accelerometric or gait features that had shown an effect in the previous analysis had a relevant predictive accuracy for episodes of disorientation. We used the area under the ROC curve to estimate a single feature’s ability to predict disorientation at a time interval. ROC analysis was done using the library “ROCnReg” in R allowing for Bayesian estimates of credibility intervals for the areas under the ROC curves.

Third (A2.3), we used a multivariate approach to find a combination of accelerometric or gait features that may contribute to relevant accuracy in the detection of disorientation episodes. In this study, we used as the primary model a support vector machine (SVM), implemented using the R library “e1071.” Before SVM training, we used feature selection based on the correlation coefficient of every single predictive feature with the dependent binary variable “oriented” *vs*. “disoriented.” Only features with an absolute value of the correlation coefficient larger than 0.12 were entered into the SVM training. After visual inspection of the data revealed no linear separation between groups, we decided to use a radial kernel whose parameters cost and gamma function were determined using a 10-fold cross-validation using the function tune in library “e1071.” To account for the binding of the data within patients, we determined the accuracy of the SVM models using patients as folds. Within each patient, 80% of each patient’s data were used as training data and the remaining 20% as test data. Accuracy was determined as the percentage of correctly classified time intervals where the predicted states of orientation or disorientation agreed with the observed states of orientation or disorientation relative to all observations per patient.

Finally (A2.4), we used a Gaussian Hidden Markov Model (HMM) respecting the temporal nature of the data. Using the HMM approach, we generated states (constraining the model to two possible states only) from the observed response variables, and subsequently compared the distribution of the generated states with the distribution of the observed states. This analysis was conducted using library “depmixS4” in R. Taking into account the origin of the time-series data, we split the analysis according to participants. We estimated transition matrices and means and standard deviations of the response variables from the data and used these estimates to fit the states’ model per participant.

## Results

Demographic characteristics of our sample can be found in Table 1. Bayes factor analysis suggested no evidence in favor of a difference in sex distribution and education years across the groups and was in favor of no difference in age between experimental and control conditions within the young and older groups, respectively. By design, young and older groups differed in age. Participants in the experimental condition were presented with altered landmarks to induce disorientation, whereas participants in the control condition were not.

**TABLE 1:** Demographic, orientation, and gait characteristics.

	Young controls	Young experimental	Older controls	Older experimental
N (f/m)^1^	4/6	8/7	9/5	9/5
Age^2^ (years) (SD)	24.2 (2.7)	24.7 (4.3)	69.5 (4.0)	72.0 (5.3)
Education^3^ (years) (SD)	13.3 (0.9)	14.0 (1.5)	13.9 (2.9)	15.0 (2.5)
Mean number disorientation^4^ (SD)	0.30 (0.95)	2.20 (2.57)	0.57 (1.09)	5.79 (3.22)
Mean accelerometry^5^ ankle (SD)	1.49 (0.11)	1.37 (0.01)	1.34 (0.10)	1.28 (0.05)
Mean walking speed^6^ (SD)	1.44 (0.16)	1.23 (0.17)	1.06 (0.21)	0.93 (0.13)

*^1^Bayes factor in favor of no difference between groups, BF_10_ = 0.157.*

*^2^Bayes factor in favor of no difference, BF_10_ = 0.736, between older experimental and control cases, and in favor of no difference, BF_10_ = 0.390, between young experimental and control cases.*

*^3^Bayes factor in favor of no difference between groups, BF_10_ = 0.341.*

*^4^Bayes factor in favor of a difference between older controls and older experimentals, older experimentals and both young controls and young experimentals (BF_10_ > 14.7).*

*^5^Bayes factor in favor of a difference between older controls and young experimentals, older experimentals and young controls, and young experimentals and young controls (BF_10_ > 9.0).*

*^6^Bayes factor in favor of a difference between older controls and young controls, older experimentals and young experimentals and young controls, and young experimentals and young controls (BF_10_ > 9.0).*

### Aggregated Data

**FIGURE 1:**
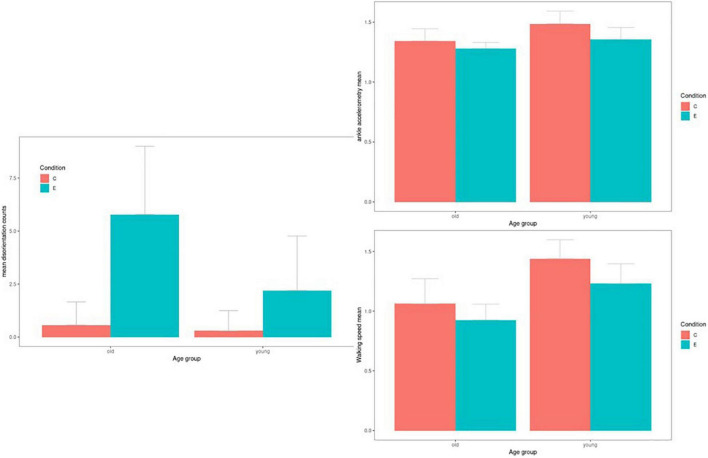
Aggregated disorientation events, accelerometry, and walking speed by age group by condition. Disorientation events (upper row), ankle worn accelerometric signal (middle row), and walking speed mean (lower row) according to age group and condition [control (C) *vs*. experimental (E)]. Bars show mean and 95% credibility intervals.

The average number of disorientation events, mean ankle-worn accelerometric signal, and walking speed per age group and the experimental condition is plotted in Figure 1. We found extreme evidence in favor of a difference between older control and experimental cases and between older experimental and young control cases, and moderate evidence in favor of a difference between older experimental and young experimental cases. Evidence for differences within the young age group and between the older control and the young control groups was not conclusive. For ankle-worn accelerometry and walking speed, there was mainly an age effect and a less-pronounced effect of experimental condition (see Table 1 for details).

Leave-one-out-cross-validation of the *Watanabe-Akaike information criterion* (WAIC) (Vehtari et al., 2017) confirmed that the Poisson regression was superior to the Gaussian regression model fit [WAIC difference in favor of Poisson = –21.6 (SE = 8.9)] when using condition, age, and gender as the only predictors for the base model.

*The number of disorientation events across the experiment* were associated with **executive function** (smaller number of disorientation counts with higher executive function), but not with visuospatial constructional ability, visual memory, or perspective-taking/spatial orientation. **Ankle-worn sensor overall level of activity** was associated with counts of disorientation (more activity, less disorientation), but not wrist or chest-worn sensors.

When considering gait features, slower **walking speed** and lower **step length** were associated with a higher number of disorientation events.

Across all models, **experimental condition** and higher **age** were associated with a higher number of disorientation events, whereas gender was unrelated to disorientation events.

Detailed results can be found in Table 2. When repeating these analyses with the percentage of disorientation events per patient’s time of experiment as an outcome, the results were essentially unchanged (data not shown). The only difference was that in addition to the previous effects, a higher wrist-worn accelerometric signal was associated with a higher percentage of disorientation events (main effect = 5.50, 95% credibility interval 2.25–8.67) as well.

**TABLE 2:** Number of disorientation events by cognitive, accelerometric, and gait features.

Cognitive scores				
**Independent variables**	**Main effect cognitive score**	**Condition**	**Age (years)**	**Gender**
**Visuospatial**	0.01 (–0.25 to 0.29)	**2.26 (1.56** to **3.05)**	**0.04 (0** to **0.08)**	–0.04 (–0.5 to 0.4)
**Executive function**	**–0.2 (–0.4** to **0.01)**	**2.2 (1.49** to **3.01)**	**0.06 (0.01** to **0.11)**	–0.09 (–0.53 to 0.34)
**Visual memory**	–0.15 (–1.22 to 1)	**2.29 (1.57** to **3.11)**	**0.04 (0** to **008)**	–0.03 (–0.47 to 0.37)
**PTSOT**	–0.12 (–0.82 to 0.48)	**2.26 (1.52** to **3.07)**	0.02 (–0.03 to 0.07)	0.1 (–0.47 to 0.67)

**Accelerometric features**				
**Independent variables**	**Main effect accelerometry**	**Condition**	**Age (years)**	**Gender**

**Ankle mean (g)**	**–4.62 (–7.66** to **–1.73)**	**1.97 (1.34** to **2.63)**	**0.01 (0.01** to **0.02)**	0.01 (–0.37 to 0.38
**Wrist mean (g)**	3.02 (–1.79 to 7.52)	**2.25 (1.68** to **2.93)**	**0.02 (0.01** to **0.03)**	–0.22 (–0.64 to 0.2)
**Chest mean (g)**	1.01 (–11.18 to 13.17)	**2.23 (1.63** to **2.92)**	**0.02 (0.01** to **0.03)**	–0.08 (–0.47 to 0.31)

**Gait features**				
**Independent variables**	**Main effect gait**	**Condition**	**Age (years)**	**Gender**

**Walking speed (m/s)**	**–2.23 (–3.46** to **–0.99)**	**1.97 (1.38** to **2.63)**	**0.01 (0** to **0.02)**	0.08 (–0.3 to 0.44)
**Step length (m)**	**–2.85 (–5.45** to **–0.31)**	**2.04 (1.44** to **2.75)**	**0.01 (0** to **0.02)**	0 (–0.38 to 0.37)
**Stride time (s)**	–0.06 (–1.08 to 0.87)	**2.24 (1.61** to **2.96)**	**0.02 (0.01** to **0.03)**	–0.09 (–0.48 to 0.31)
**Step width (m)**	0.93 (–4.56 to 6.32)	**2.22 (1.63** to **2.9)**	**0.02 (0.01** to **0.03)**	–0.12 (–0.56 to 0.3)
**Stance time (s)**	0.43 (–0.73 to 1.56)	**2.33 (1.71** to **3.12)**	**0.02 (0.01** to **0.03)**	–0.04 (–0.43 to 0.33)
**Swing time (s)**	–2.78 (–5.91 to 0.11)	**2.11 (1.5** to **2.8)**	**0.02 (0.01** to **0.03)**	–0.15 (–0.52 to 0.22)

*Gender = factor level effects for male vs. female sex.*

*Cognitive variables represent domain scores derived as the mean score of specific tests, after transformation to z-scores.*

*Values in bold indicate effects where the 95% credibility interval excludes 0.*

*g = acceleration constant g (1 g = 9.81 m/s^2^).*

*m = meter.*

*s = seconds.*

### Real-Time Data

**FIGURE 2:**
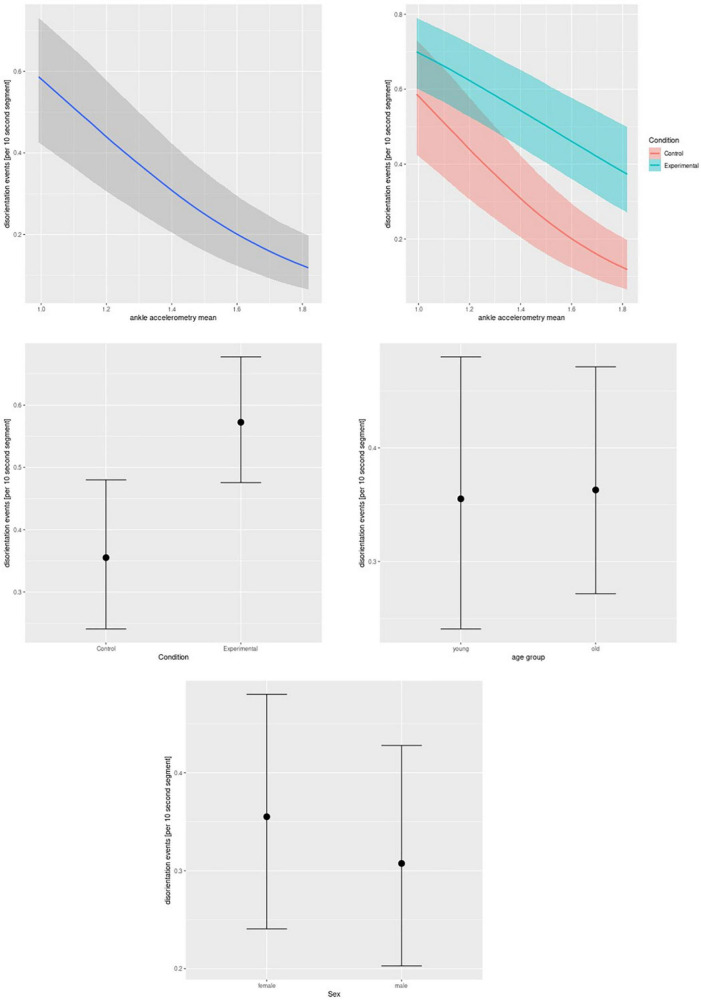
Real-time data, ankle worn accelerometry. Bayesian mixed-effect logistic regression of disorientation events on ankle worn accelerometric signal (main effect, upper left and interaction effect with condition, upper right), condition (experimental or control, middle left), age group (middle right), and gender (lower row). The graphs feature mean effects and 95% credibility intervals.

For **accelerometric features**, we found the main effect of lower ankle, wrist, and chest-worn sensors’ levels of activity with more disorientation events. In addition, we found interactions of ankle- and wrist-worn sensors’ levels of activity with the experimental condition, showing more pronounced negative associations in the control than in the experimental condition (see Figure 2 for an example of ankle-worn sensor activity). In addition, experimental condition, but not age or gender, was associated with more disorientation events. See Table 2 for details.

**FIGURE 3:**
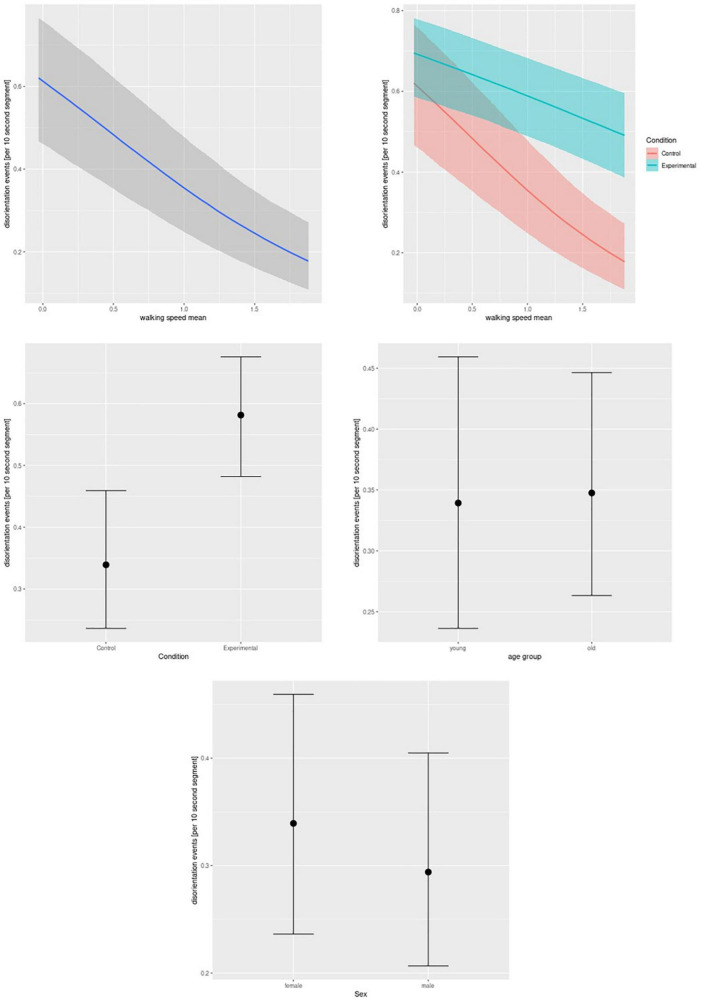
Real-time data, walking speed. Bayesian mixed-effect logistic regression of disorientation events on mean walking speed (main effect, upper left and interaction effect with condition, upper right), condition (experimental or control, middle left), age group (middle right), and gender (lower row). The graphs feature mean effects and 95% credibility intervals.

For **gait features**, all *a priori* selected gait features showed a main effect on disorientation events. Lower walking speed and step length and width as well as longer stride, swing, and stance times were associated with more disorientation events. In addition, we found interactions of walking speed, step length, step width, and swing time with an experimental condition, showing more pronounced negative associations in the control than the experimental condition for walking speed, step length, and step width, and a more positive association for swing time (see Figure 3 for an example of walking speed). In addition, experimental condition, but not age or gender, was associated with more disorientation events. See Table 3 for details.

**TABLE 3:** Incidence of disorientation events and accelerometric and gait features in real time.

Accelerometric features					
**Independent variables**	**Main effect**	**Accelerometry by Condition**	**Condition**	**Age group**	**Gender**
**Ankle mean**	**–0.48 (–0.64** to **–0.32)**	**0.2 (0.02** to **0.38)**	**0.93 (0.44** to **1.39)**	0.04 (–0.45 to 0.49)	–0.19 (–0.65 to 0.26)
**Wrist mean**	**–0.67 (–0.91** to **–0.43)**	**0.27 (0.01** to **0.54)**	**1.21 (0.78** to **1.65)**	–0.16 (–0.61 to 0.29)	–0.03 (–0.47 to 0.4)
**Chest mean**	**–0.45 (–0.68** to **–0.24)**	0.18 (–0.08 to 0.43)	**0.99 (0.53** to **1.46)**	–0.23 (–0.76 to 0.28)	–0.21 (–0.66 to 0.24)

**Gait features**					
**Independent variables**	**Main effect**	**Gait by Condition**	**Condition**	**Age group**	**Gender**

**Walking speed**	**–0.47 (–0.62** to **–0.32)**	**0.27 (0.1** to **0.44)**	**1 (0.56** to **1.44)**	0.04 (–0.44 to 0.5)	–0.2 (–0.64 to 0.22)
**Step length**	**–2.69 (–3.5** to **–1.85)**	**2.08 (1.13** to **2.99)**	**1.07 (0.62** to **1.52)**	0.07 (–0.35 to 0.52)	–0.21 (–0.67 to 0.22)
**Stride time**	**0.19 (0.11** to **0.29)**	–0.03 (–0.21 to 0.21)	**1.17 (0.72** to **1.61)**	0.27 (–0.16 to 0.7)	–0.29 (–0.7 to 0.12)
**Step width**	**–0.71 (–0.95** to **–0.5)**	**0.79 (0.55** to **1.04)**	**1.36 (0.92** to **1.84)**	0.21 (–0.24 to 0.67)	–0.32 (–0.78 to 0.14)
**Stance time**	**0.14 (0.06** to **0.22)**	0.05 (–0.14 to 0.28)	**1.18 (0.73** to **1.61)**	0.25 (–0.16 to 0.67)	–0.32 (–0.73 to 0.12)
**Swing time**	**0.34 (0.19** to **0.52)**	**–0.27 (–0.5** to **–0.04)**	**1.17 (0.74** to **1.61)**	0.27 (–0.17 to 0.7)	–0.3 (–0.74 to 0.14)

*Age group = old vs. young.*

*Gender = factor level effects for male vs. female sex.*

*The accelerometric and gait variables were z-score transformed before being entered into the models.*

*Values in bold indicate effects where the 95% credibility interval excludes 0.*

### Accuracy of Disorientation Event Detection

We used the Bayesian ROC curve analysis to estimate the accuracy of single markers that had shown an association with orientation in the previous mixed-effect models. For ankle-worn accelerometric signal, the area under the ROC curve was 0.60 (95% credibility interval 0.588–0.615). For the remaining accelerometric features and the gait features, AUC values were below 0.60. These number indicate a detectable, but clinically irrelevant effect of single markers on accuracy levels.

**FIGURE 4:**
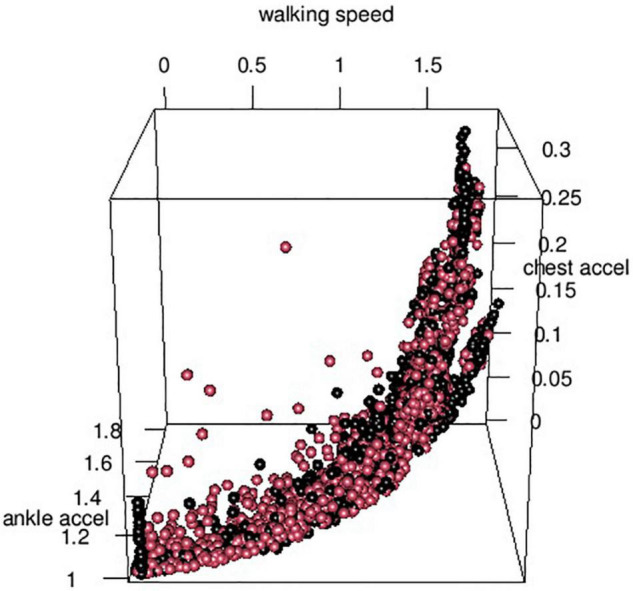
Distribution of orientation status across features. Three-dimensional representation of the distribution of orientation status (oriented – black beads, disoriented – red beads) across ankle and chest-worn accelerometric signal and walking speed mean.

Subsequently, we implemented a multivariate cross-validated support vector machine to determine the accuracy of a (non-linear combination of markers). Feature selection was done using absolute correlation coefficients > 0.12 between candidate features and orientation status across all data. We chose a radial kernel as the plotting of data did not indicate a linear separation (see Figure 4), with a cost parameter of 10 and a gamma parameter of 1, based on the initial grid search using the whole data set. Subsequently, we determined group discrimination within each patient fold applied to a random selection of 80% of the data as a training sample and the remaining 20% of data as a test sample. The mean accuracy of correctly allocated instances of orientation/disorientation was 72% (SD 11%) across the cross-validated patient folds.

**FIGURE 5:**
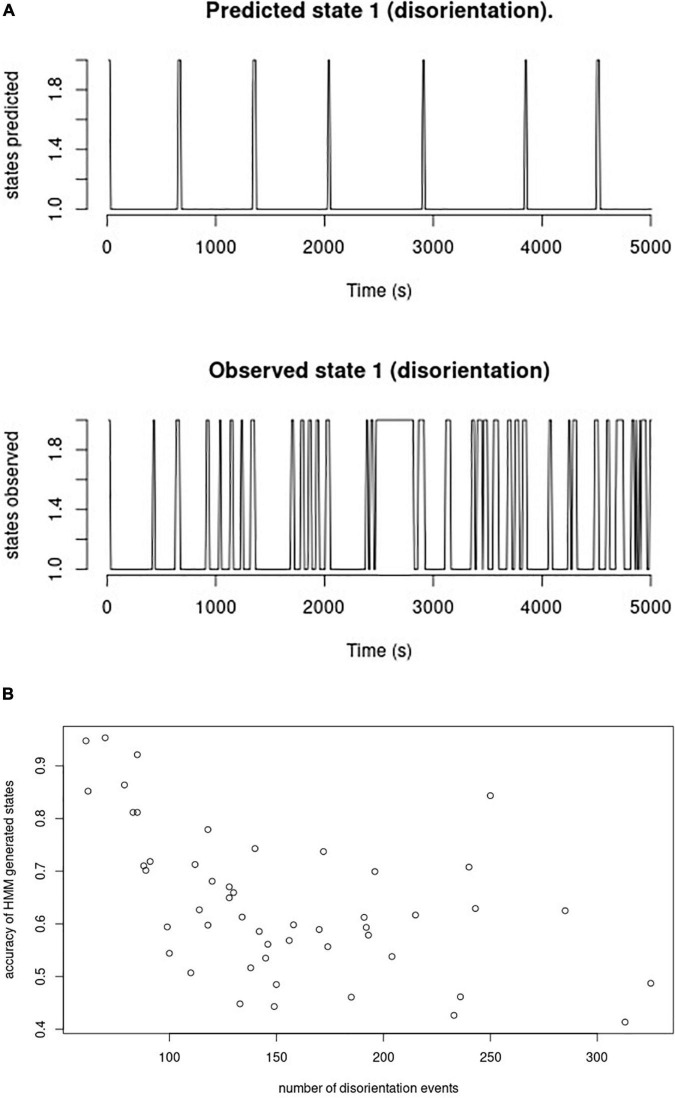
Hidden Markov model generated states and observed orientation states. **(A)** Time series of states within 5,000 s. The upper row plots the orientation states generated from the Hidden Markov model during the first 500 time segments (= 5,000 s, pooled across participants) with 1 = oriented, 2 = disoriented; the lower row plots the observed orientation states from the same time segments. **(B)** Association between number of disorientation events and accuracy of HMM generated states. This graph plots the accuracy of the HMM generated states relative to the observed states per participant (y-axis) vs. the number of disorientation events per participant (x-axis).

Using a generative Hidden Markov model implemented in library “depmixS4” in R reached an average accuracy of correctly allocated instances of orientation/disorientation of only 64% (SD 14%) when comparing the binary states of oriented/disoriented as generated from the observed variables ankle-worn accelerometric signal and walking speed mean and variance as compared with the observed disorientation instances. As can be seen from Figure 5, the Hidden Markov model produced substantially fewer disorientation states than had been observed (Figure 5A), and accuracy decreased with a higher number of observed disorientation states per patient (Figure 5B), with a correlation coefficient of –0.51.

## Discussion

Here, we studied the association of accelerometric and gait features with episodes of disorientation in cognitively normal young and older adults in a hybrid experiment. We found that decreased accelerometric signal from ankle-worn sensors as well as decrease in walking speed and step length were associated with a higher number of aggregated disorientation events. Similarly, decreases in accelerometric signal and changes in a range of spatiotemporal gait features were associated with a higher number of episodes of disorientation in real time. At the same time, the prediction accuracy of single accelerometric and gait features for episodes of disorientation in real time was below 60%. However, even when combining the most strongly associated features in a multivariate non-linear support vector machine, reached only 72% accuracy for correctly allocated instances of orientation/disorientation. This level of accuracy would not be sufficient for individual detection of disorientation episodes and situation-aware assistance. Thus, we were able to confirm the expected association of accelerometric and gait characteristics with disorientation in cognitively unimpaired individuals, but we did not find sufficient accuracy for individual prediction.

Our study was able to replicate the age-related decline in spatiotemporal gait features that has been reported in a large number of studies, systematically reviewed in Herssens et al. (2018) and Osoba et al. (2019). Spatial orientation requires visuospatial abilities and higher-order cognitive processes, such as egocentric and allocentric representations, cognitive mapping, spatial strategies, encoding, and processing of spatial information (Lithfous et al., 2013; Meneghetti et al., 2014; Muffato et al., 2016). In our study, we focused on the domains of visual memory, visuoconstructional ability, executive function, and spatial orientation. Our results demonstrated a relationship between executive function and aggregated orientation in older adults; the number of disorientation events was lower in individuals with higher executive function. In this study, we had used the ratio of Trail Making Test B to A as a measure of executive function, assessing motor speed and visual speed (Arbuthnott and Frank, 2000; Sanchez-Cubillo et al., 2009). The Trail Making Test ratio serves as an index of executive control function because it can provide an independent measure of cognitive flexibility (Bezdicek et al., 2017). Moreover, it has also been associated with frontal executive function (Arbuthnott and Frank, 2000). An association of executive functions and effective spatial navigation has been previously reported (Wei et al., 2020; Laczo et al., 2021). Based on our results, we assume that higher executive functions play an important role in tasks requiring the use of effective wayfinding strategies. Effects on visuospatial abilities were absent, whereas effects on visual memory were not conclusive. We had expected an association between these domains, since they have been implicated in navigation efficiency and environment learning (Meneghetti et al., 2014; Wei et al., 2020). Previous studies have demonstrated an age-related decline in navigation skills, due to difficulties in environment route learning and spatial recall of relationships between landmarks and directions at decisions points (Zhong and Moffat, 2016; Ramanoel et al., 2020). The absence of an effect, therefore, was unexpected. A post hoc explanation would relate to previous observations that paper–pencil testing of spatial abilities found a poor correlation with real-world navigation performance (Nadolne and Stringer, 2001; Taillade et al., 2015), which has been used as an argument for the creation of novel ecologically valid test instruments (Nadolne and Stringer, 2001).

Furthermore, we had expected an association of orientation with the Perspective Taking/Spatial Orientation Test, since previous work suggested alterations of egocentric topographic orientation in older adults (Caffo et al., 2020). Two of the 28 participants, however, were not able to perform the task at all and several participants had difficulties when performing the Perspective Taking/Spatial Orientation Test. As we saw in practice, it was challenging for our participants to understand the task instructions and they might have felt overstrained. Difficulties regarding the understanding of instructions on similar tasks have been previously reported in young adults (Hegarty and Waller, 2004). Although the Perspective Taking/Spatial Orientation Test by design seemed well suited to test a trait of orientation ability and it has been widely used in spatial cognition literature (Friedman et al., 2020), it was not easy to use, at least in our hands, even for cognitively normal older people. The test has only been used in a few previous studies with older people (Zancada-Menendez et al., 2016) who on average were 8–10 years younger than our older group of participants.

A relationship between gait characteristics and disorientation has already been demonstrated in conditions such as delirium and dementia (Arjunan et al., 2019; Evensen et al., 2019; Oh, 2021; Weizman et al., 2021). In contrast, the detection of disorientation events using gait and accelerometry features has been little explored. In a similar set-up to our study, one previous study reported gait features for a group of 17 young and 17 older participants navigating on a treadmill through a virtual shopping mall (Kafri et al., 2021). However, detection of disorientation was not an outcome parameter in this earlier study. In the current study, we found that reduced ankle-worn accelerometry signal was associated with more disorientation events in both aggregated and real-time data. The reduction of walking speed and step length and the increase in stance, swing, and stride time was associated with more disorientation events. This is consistent with the reduction in overall signal from the ankle-worn sensors and suggests that the acceleration signal may be useful as a surrogate measure for less easily measured gait characteristics, but with the caveat that none of the gait characteristics examined achieved a useful level of predictive accuracy for disorientation events.

Even when combining features in a non-linear support vector machine, the accuracy level in our hybrid set-up was below the accuracy level which we had achieved in a real-world experiment with people with MCI or dementia. In this study, the accelerometric features had achieved an AUC of 75% and 79% of correctly allocated instances of orientation/disorientation (Schaat et al., 2019). In the previous experiment, we struggled with the low occurrence of disorientation episodes relative to the total time of the experiment, which made training the models difficult and led to unbalanced sensitivity and specificity estimates. In this study, we wanted to improve this situation in a much more controlled environment. Based on this setting, we were able to focus on the time series at the decision points only and induce disorientation even in young individuals. Indeed, this approach was successful with a proportion of 41% of intervals being annotated as disorientated in the total time series, and 49% at the decision points compared with less than 10% in the previous real-world setting (Schaat et al., 2019). Although we achieved a higher proportion of disorientation events our models performed less accurately. There are several post hoc explanations for this unexpected result which also relate to the limitations of our study.

The limitations of our study include the following points: First, the measurement of orientation states was based on offline video annotation which carries some imprecision. However, inter-rater reliability was very good (Cohen’s kappa > 0.8), and even using lagged features, allowing sensor values of a time frame of 30 s before the actual rating of disorientation to be included in the prediction models, did not alter the results. Second, the difference in set-up where walking on a treadmill and walking on a street pose different requirements on cognitive and motion abilities so that the resulting gait and movement features may not directly be comparable. A previous study reported a slower gait with shorter, less variable strides during treadmill walking compared with walking outdoors on the sideway in young and older adults (Schmitt et al., 2021). Thus, walking on a motorized treadmill may reduce the variability of gait characteristics compared with walking outdoors, thereby also reducing disorientation-induced changes in gait characteristics. Third, in this study, we had studied cognitively unimpaired individuals who may show less pronounced changes in walking behavior during episodes of disorientation than individuals with MCI or dementia who were lost in a real-world setting (Schaat et al., 2019). Fourth, from the Hidden Markov model, it became obvious that the model produced less instances of disorientation than were observed, that is, only approximately 64%. In comparison, the previous model for the real-world data had produced a high number of false alarms, that is, more instances of disorientation than had been observed (Schaat et al., 2019). This may suggest that grouping disorientation events into only two states (oriented *vs*. not oriented) was too simplistic for the present data. There may be different subtypes of disorientation states, each associated with different behavioral characteristics. For example, externally triggered disorientation events might represent a different category of disorientation states than spontaneously occurring disorientation events; however, the two states were not distinguished in our models. Finally, the sample size was relatively small in our study. Consequently, our study was only powered to detect moderate-to-large effects. The effort required to complete the experiment was high for each participant. So we had even considered to use a cross-over design where each participant would undergo both conditions, experimental and control, in a randomized, balanced design. We decided against this option because already the experiment with only one condition was exhausting for some of the older participants.

In summary, in a prospective analysis of young and older cognitively healthy adults in a hybrid environment featuring a treadmill-based navigation through a virtual environment, we found an association between executive function, ankle-worn accelerometric signal, and spatiotemporal gait features with an aggregated number of disorientation events across age groups and experimental conditions. This was replicated by an association of accelerometric signal and spatiotemporal gait features with disorientation events in the real-time data analysis. Despite these consistent associations, the predictive accuracy of single or combined acceleration and gait features was insufficient for individual detection of disorientation events in real time. The lessons from this analysis are that age-related and experimentally induced disorientation is reflected in spatiotemporal gait features and also in the accelerometric signal as a potentially more easily accessible surrogate for gait features. At the same time, such measurements probably need to be enriched with other parameters to be sufficiently accurate for individual prediction of disorientation events. In future directions, further experiments may test whether such predictions can be more accurate for people with dementia. For this group of individuals, based on our preliminary experience with a small number of patients, external induction of disorientation events is not necessary, as they already showed pronounced disorientation under undisturbed control conditions. Finally, the set-up of our experiment may be useful not only to monitor but even to train navigation abilities under dual-task conditions with high transfer potential to real-world environment.

## Data Availability Statement

The raw data supporting the conclusions of this article will be made available by the authors, without undue reservation.

## Ethics Statement

The studies involving human participants were reviewed and approved by Ethics committee of the Rostock University Medical Center (Approval No. A 2019-0062). The patients/participants provided their written informed consent to participate in this study.

## Author Contributions

ST was involved in all stages of the work, contributing to the study design, research question, performed analyses and interpretation of the data, and drafted and revised the manuscript. CA and SL contributed to the acquisition of the data, provided feedback and revised the manuscript. DG provided feedback and revised the manuscript. SF contributed to acquisition of the neuropsychological data, provided feedback, and revised the manuscript. SB provided feedback and revised the manuscript. TK contributed significantly to the conception and design of the study, provided feedback, and revised the manuscript. All authors read and approved the manuscript.

## Conflict of Interest

ST participated in scientific advisory boards of Roche Pharma AG, Biogen, GRIFOLS, EISAI, and MSD and received lecture fees from Roche and MSD. The remaining authors declare that the research was conducted in the absence of any commercial or financial relationships that could be construed as a potential conflict of interest.

## Publisher’s Note

All claims expressed in this article are solely those of the authors and do not necessarily represent those of their affiliated organizations, or those of the publisher, the editors and the reviewers. Any product that may be evaluated in this article, or claim that may be made by its manufacturer, is not guaranteed or endorsed by the publisher.

## References

[B1] Albert M. S. Dekosky S. T. Dickson D. Dubois B. Feldman H. H. Fox N. C. (2011). The diagnosis of mild cognitive impairment due to Alzheimer’s disease: recommendations from the National Institute on Aging-Alzheimer’s Association workgroups on diagnostic guidelines for Alzheimer’s disease. *Alzheimers Dement.* 7 270–279. 10.1016/j.jalz.2011.03.008 21514249 PMC3312027

[B2] Amaefule C. O. Ludtke S. Kirste T. Teipel S. J. (2020). Effect of spatial disorientation in a virtual environment on gait and vital features in patients with dementia: pilot single-blind randomized control trial. *JMIR Serious Games* 8:e18455. 10.2196/18455 33030436 PMC7582144

[B3] Arbuthnott K. Frank J. (2000). Trail making test, part B as a measure of executive control: validation using a set-switching paradigm. *J. Clin. Exp. Neuropsychol.* 22 518–528. 10.1076/1380-3395(200008)22:4;1-0;FT518 10923061

[B4] Arjunan A. Peel N. M. Hubbard R. E. (2019). Gait speed and frailty status in relation to adverse outcomes in geriatric rehabilitation. *Arch. Phys. Med. Rehabil.* 100 859–864. 10.1016/j.apmr.2018.08.187 30312596

[B5] Baudendistel S. T. Schmitt A. C. Stone A. E. Raffegeau T. E. Roper J. A. Hass C. J. (2021). Faster or longer steps: maintaining fast walking in older adults at risk for mobility disability. *Gait Posture* 89 86–91. 10.1016/j.gaitpost.2021.07.002 34256264 PMC9277656

[B6] Beauchet O. Allali G. Sekhon H. Verghese J. Guilain S. Steinmetz J. P. (2017). Guidelines for assessment of gait and reference values for spatiotemporal gait parameters in older adults: the biomathics and canadian gait consortiums initiative. *Front. Hum. Neurosci.* 11:353. 10.3389/fnhum.2017.00353 28824393 PMC5540886

[B7] Becu M. Sheynikhovich D. Tatur G. Agathos C. P. Bologna L. L. Sahel J. A. (2020). Age-related preference for geometric spatial cues during real-world navigation. *Nat. Hum. Behav.* 4 88–99. 10.1038/s41562-019-0718-z 31548677

[B8] Bezdicek O. Stepankova H. Axelrod B. N. Nikolai T. Sulc Z. Jech R. (2017). Clinimetric validity of the Trail Making Test Czech version in Parkinson’s disease and normative data for older adults. *Clin. Neuropsychol.* 31 42–60. 10.1080/13854046.2017.1324045 28534428

[B9] Caffo A. O. Lopez A. Spano G. Serino S. Cipresso P. Stasolla F. (2018). Spatial reorientation decline in aging: the combination of geometry and landmarks. *Aging Ment. Health* 22 1372–1383. 10.1080/13607863.2017.1354973 28726502

[B10] Caffo A. O. Lopez A. Spano G. Stasolla F. Serino S. Cipresso P. (2020). The differential effect of normal and pathological aging on egocentric and allocentric spatial memory in navigational and reaching space. *Neurol. Sci.* 41 1741–1749. 10.1007/s10072-020-04261-4 32002741

[B11] Cohen J. A. Verghese J. (2019). Gait and dementia. *Handb. Clin. Neurol.* 167 419–427. 31753146 10.1016/B978-0-12-804766-8.00022-4

[B12] Coley N. Gallini A. Ousset P. J. Vellas B. Andrieu S. Guidage Study G. (2016). Evaluating the clinical relevance of a cognitive composite outcome measure: an analysis of 1414 participants from the 5-year GuidAge Alzheimer’s prevention trial. *Alzheimers Dement.* 12 1216–1225. 10.1016/j.jalz.2016.06.002 27423962

[B13] Costa R. Pompeu J. E. Viveiro L. A. P. Brucki S. M. D. (2020). Spatial orientation tasks show moderate to high accuracy for the diagnosis of mild cognitive impairment: a systematic literature review. *Arq. Neuropsiquiatr.* 78 713–723. 10.1590/0004-282X20200043 33331465

[B14] Cushman L. A. Stein K. Duffy C. J. (2008). Detecting navigational deficits in cognitive aging and Alzheimer disease using virtual reality. *Neurology* 71 888–895. 10.1212/01.wnl.0000326262.67613.fe 18794491 PMC2676944

[B15] Dahmani L. Bohbot V. D. (2020). Habitual use of GPS negatively impacts spatial memory during self-guided navigation. *Sci. Rep.* 10:6310. 10.1038/s41598-020-62877-0 32286340 PMC7156656

[B16] Evensen S. Bourke A. K. Lydersen S. Sletvold O. Saltvedt I. Wyller T. B. (2019). Motor activity across delirium motor subtypes in geriatric patients assessed using body-worn sensors: a Norwegian cross-sectional study. *BMJ Open* 9:e026401. 10.1136/bmjopen-2018-026401 30826800 PMC6398701

[B17] Friedman A. Kohler B. Gunalp P. Boone A. P. Hegarty M. (2020). A computerized spatial orientation test. *Behav. Res. Methods* 52 799–812. 10.3758/s13428-019-01277-3 31347037

[B18] Gazova I. Vlcek K. Laczo J. Nedelska Z. Hyncicova E. Mokrisova I. (2012). Spatial navigation-a unique window into physiological and pathological aging. *Front. Aging Neurosci.* 4:16. 10.3389/fnagi.2012.00016 22737124 PMC3380196

[B19] Hegarty M. Waller D. (2004). A dissociation between mental rotation and perspective-taking spatial abilities. *Intelligence* 32 175–191. 10.1016/j.intell.2003.12.001

[B20] Herssens N. Verbecque E. Hallemans A. Vereeck L. Van Rompaey V. Saeys W. (2018). Do spatiotemporal parameters and gait variability differ across the lifespan of healthy adults? A systematic review. *Gait Posture* 64 181–190. 10.1016/j.gaitpost.2018.06.012 29929161

[B21] Iachini I. Iavarone A. Senese V. P. Ruotolo F. Ruggiero G. (2009). Visuospatial memory in healthy elderly, AD and MCI: a review. *Curr. Aging Sci.* 2 43–59. 10.2174/1874609810902010043 20021398

[B22] Kafri M. Weiss P. L. Zeilig G. Bondi M. Baum-Cohen I. Kizony R. (2021). Performance in complex life situations: effects of age, cognition, and walking speed in virtual versus real life environments. *J. Neuroeng. Rehabil.* 18:30. 10.1186/s12984-021-00830-6 33557894 PMC7871373

[B23] Kizony R. Zeilig G. Krasovsky T. Bondi M. Weiss P. L. Kodesh E. (2017). Using virtual reality simulation to study navigation in a complex environment as a functional-cognitive task; a pilot study. *J. Vestib. Res.* 27 39–47. 10.3233/VES-170605 28387691

[B24] Laczo M. Wiener J. M. Kalinova J. Matuskova V. Vyhnalek M. Hort J. (2021). Spatial navigation and visuospatial strategies in typical and atypical aging. *Brain Sci.* 11:1421. 10.3390/brainsci11111421 34827423 PMC8615446

[B25] Lester A. W. Moffat S. D. Wiener J. M. Barnes C. A. Wolbers T. (2017). The aging navigational system. *Neuron* 95 1019–1035. 10.1016/j.neuron.2017.06.037 28858613 PMC5659315

[B26] Lithfous S. Dufour A. Despres O. (2013). Spatial navigation in normal aging and the prodromal stage of Alzheimer’s disease: insights from imaging and behavioral studies. *Ageing Res. Rev.* 12 201–213. 10.1016/j.arr.2012.04.007 22771718

[B27] Lohman E. B. III Balan Sackiriyas K. S. Swen R. W. (2011). A comparison of the spatiotemporal parameters, kinematics, and biomechanics between shod, unshod, and minimally supported running as compared to walking. *Phys. Ther. Sport* 12 151–163. 10.1016/j.ptsp.2011.09.004 22085708

[B28] Mc Ardle R. Del Din S. Donaghy P. Galna B. Thomas A. J. Rochester L. (2021). The impact of environment on gait assessment: considerations from real-world gait analysis in dementia subtypes. *Sensors (Basel)* 21:813. 10.3390/s21030813 33530508 PMC7865394

[B29] McKhann G. M. Knopman D. S. Chertkow H. Hyman B. T. Jack C. R. Jr. Kawas C. H. (2011). The diagnosis of dementia due to Alzheimer’s disease: recommendations from the National Institute on Aging-Alzheimer’s Association workgroups on diagnostic guidelines for Alzheimer’s disease. *Alzheimers Dement.* 7 263–269. 10.1016/j.jalz.2011.03.005 21514250 PMC3312024

[B30] Meneghetti C. Ronconi L. Pazzaglia F. De Beni R. (2014). Spatial mental representations derived from spatial descriptions: the predicting and mediating roles of spatial preferences, strategies, and abilities. *Br. J. Psychol.* 105 295–315. 10.1111/bjop.12038 25040003

[B31] Morris J. C. Heyman A. Mohs R. C. Hughes J. P. Van Belle G. Fillenbaum G. (1989). The Consortium to Establish a Registry for Alzheimer’s disease (CERAD). Part I. Clinical and neuropsychological assessment of Alzheimer’s disease. *Neurology* 39 1159–1165. 10.1212/wnl.39.9.1159 2771064

[B32] Muffato V. Meneghetti C. De Beni R. (2016). Not all is lost in older adults’ route learning: the role of visuo-spatial abilities and type of task. *J. Environ. Psychol.* 47 230–241. 10.1016/j.jenvp.2016.07.003

[B33] Mulas I. Putzu V. Asoni G. Viale D. Mameli I. Pau M. (2021). Clinical assessment of gait and functional mobility in Italian healthy and cognitively impaired older persons using wearable inertial sensors. *Aging Clin. Exp. Res.* 33 1853–1864. 10.1007/s40520-020-01715-9 32978750 PMC7518096

[B34] Nadolne M. J. Stringer A. Y. (2001). Ecologic validity in neuropsychological assessment: prediction of wayfinding. *J. Int. Neuropsychol. Soc.* 7 675–682. 10.1017/s1355617701766039 11575589

[B35] Oh C. (2021). Single-task or dual-task? gait assessment as a potential diagnostic tool for Alzheimer’s dementia. *J. Alzheimers Dis.* 84 1183–1192. 10.3233/JAD-210690 34633320 PMC8673517

[B36] Osoba M. Y. Rao A. K. Agrawal S. K. Lalwani A. K. (2019). Balance and gait in the elderly: a contemporary review. *Laryngoscope Investig. Otolaryngol.* 4 143–153. 10.1002/lio2.252 30828632 PMC6383322

[B37] Osterrieth P. A. (1944). Le test de copie d’une figure complexe; contribution à l’étude de la perception et de la mémoire [Test of copying a complex figure; contribution to the study of perception and memory]. *Arch. Psychol.* 30 206–356.

[B38] Paliokas I. Kalamaras E. Votis K. Doumpoulakis S. Lakka E. Kotsani M. (2020). Using a virtual reality serious game to assess the performance of older adults with frailty. *Adv. Exp. Med. Biol.* 1196 127–139. 10.1007/978-3-030-32637-1_13 32468314

[B39] Panel on Prevention of Falls in Older Persons, American Geriatrics Society and British Geriatrics Society (2011). Summary of the Updated American Geriatrics Society/British Geriatrics Society clinical practice guideline for prevention of falls in older persons. *J. Am. Geriatr. Soc.* 59 148–157. 10.1111/j.1532-5415.2010.03234.x 21226685

[B40] Pawlaczyk N. Szmytke M. Meina M. Lewandowska M. Stepniak J. Balaj B. (2021). Gait analysis under spatial navigation task in elderly people-a pilot study. *Sensors (Basel)* 21:270. 10.3390/s21010270 33401584 PMC7796419

[B41] Ramanoel S. Durteste M. Becu M. Habas C. Arleo A. (2020). Differential brain activity in regions linked to visuospatial processing during landmark-based navigation in young and healthy older adults. *Front. Hum. Neurosci.* 14:552111. 10.3389/fnhum.2020.552111 33240060 PMC7668216

[B42] Rey A. (1941). L’examen psychologique dans les cas d’encephopathie traumatique (The psychological examination of cases of traumatic encephalopathy). *Arch. Psychol.* 28 286–340.

[B43] Sanchez-Cubillo I. Perianez J. A. Adrover-Roig D. Rodriguez-Sanchez J. M. Rios-Lago M. Tirapu J. (2009). Construct validity of the Trail Making Test: role of task-switching, working memory, inhibition/interference control, and visuomotor abilities. *J. Int. Neuropsychol. Soc.* 15 438–450. 10.1017/S1355617709090626 19402930

[B44] Schaat S. Koldrack P. Yordanova K. Kirste T. Teipel S. (2019). Real-time detection of spatial disorientation in persons with mild cognitive impairment and dementia. *Gerontology* 66 85–94. 10.1159/000500971 31362286

[B45] Schmitt A. C. Baudendistel S. T. Lipat A. L. White T. A. Raffegeau T. E. Hass C. J. (2021). Walking indoors, outdoors, and on a treadmill: gait differences in healthy young and older adults. *Gait Posture* 90 468–474. 10.1016/j.gaitpost.2021.09.197 34619613

[B46] Taillade M. N’kaoua B. Sauzeon H. (2015). Age-related differences and cognitive correlates of self-reported and direct navigation performance: the effect of real and virtual test conditions manipulation. *Front. Psychol.* 6:2034. 10.3389/fpsyg.2015.02034 26834666 PMC4725096

[B47] Tascon L. Castillo J. Leon I. Cimadevilla J. M. (2018). Walking and non-walking space in an equivalent virtual reality task: sexual dimorphism and aging decline of spatial abilities. *Behav. Brain Res.* 347 201–208. 10.1016/j.bbr.2018.03.022 29555340

[B48] Teipel S. Babiloni C. Hoey J. Kaye J. Kirste T. Burmeister O. K. (2016). Information and communication technology solutions for outdoor navigation in dementia. *Alzheimers Dement.* 12 695–707. 10.1016/j.jalz.2015.11.003 26776761

[B49] Vehtari A. Gelman A. Gabry J. (2017). Practical Bayesian model evaluation using leave-one-out cross-validation and WAIC. *Stat. Comput.* 27 1413–1432. 10.1007/s11222-016-9696-4

[B50] Voss T. Li J. Cummings J. Farlow M. Assaid C. Froman S. (2018). Randomized, controlled, proof-of-concept trial of MK-7622 in Alzheimer’s disease. *Alzheimers Dement. (N Y)* 4 173–181. 10.1016/j.trci.2018.03.004 29955661 PMC6021552

[B51] Wei E. X. Anson E. R. Resnick S. M. Agrawal Y. (2020). Psychometric tests and spatial navigation: data from the baltimore longitudinal study of aging. *Front. Neurol.* 11:484. 10.3389/fneur.2020.00484 32595588 PMC7300262

[B52] Weizman Y. Tirosh O. Beh J. Fuss F. K. Pedell S. (2021). Gait assessment using wearable sensor-based devices in people living with dementia: a systematic review. *Int. J. Environ. Res. Public Health* 18:12735. 10.3390/ijerph182312735 34886459 PMC8656771

[B53] Wittenburg P. Brugman H. Russel A. Klassmann A. Sloetjes H. (2006). “Elan: a professional framework for multimodality research,” in *Proceedings of the 5th International Conference on Language Resources and Evaluation.* (Genoa).

[B54] Yordanova K. Koldrack P. Heine C. Henkel R. Martin M. Teipel S. (2017). Situation model for situation-aware assistance of dementia patients in outdoor mobility. *J. Alzheimers Dis.* 60 1461–1476. 10.3233/JAD-170105 29060937 PMC5676980

[B55] Zakzanis K. K. Quintin G. Graham S. J. Mraz R. (2009). Age and dementia related differences in spatial navigation within an immersive virtual environment. *Med. Sci. Monit.* 15 CR140–CR150. 19333197

[B56] Zancada-Menendez C. Sampedro-Piquero P. Lopez L. Mcnamara T. P. (2016). Age and gender differences in spatial perspective taking. *Aging Clin. Exp. Res.* 28 289–296. 10.1007/s40520-015-0399-z 26138819

[B57] Zhong J. Y. Moffat S. D. (2016). Age-related differences in associative learning of landmarks and heading directions in a virtual navigation task. *Front. Aging Neurosci.* 8:122. 10.3389/fnagi.2016.00122 27303290 PMC4882336

