# Supplementary material for: Prediction of Disorientation by Accelerometric and Gait Features in Young and Older Adults Navigating in a Virtually Enriched Environment
Source: Front Psychol. 2022 Apr 25;13:882446. doi: 10.3389/fpsyg.2022.882446 (PMC9083357; doi:10.3389/fpsyg.2022.882446)
Supplement: Supplementary file 1 [file Table_1.docx]

**Supplementary Table 1. A priori defined feature sets**

**Available classes of features (undergoing feature selection for real time classification models)**

- Accelerometric features (mean and varianace of signal within time interval, and at lagged time intervals**)**
  - Ankle worn
  - Wrist worn
  - Chest worn
- Gait features (mean, variance, and coefficient of variation within time interval, and at lagged time intervals)
  - Walking speed
  - Step length
  - Step width
  - Stride time
  - Swing time
  - Stance swing
  - Stance time
  - Trunc features (tilt, flecaton, rotation)
  - Pelvic features (tilt, oblique, rotation)
  - Ankle, hip, knee features (flexion/extension, abduction/adduction, rotation)

**A priori selected subset for aggregated models and real time mixed effect models**

- Mean accelerometric signal
  - Ankle worn
  - Wrist worn
  - Chest worn
- Mean of Gait features
  - Walking speed
  - Step length,
  - Stride time
  - Step width
  - Stance time
  - Swing time mean

**A priori selected subset for generative Markov model**

- Mean accelerometric signal
  - Ankle worn
- Mean of Gait features
  - Walking speed

**Supplementary Table 2: Proportion of disorientation events at decision points**

- DP1 0.51
  - DP1a 0.71
  - DP1b 0.65
- DP2 0.41
  - DP2a 0.06
  - DP2b 0.01
- DP3 0.13
- DP4 0.56
- DP5 0.44
  - DP5a 0.667
- DP6 0.37
  - DP6a 0.21
- DP7 0.57
  - DP7a 0.86
  - DP7b 0.89
- DP8 0.45
  - DP8a 0.39
- DP9 0.45
  - DP9a 0.81
- DP10 0.63
  - DP10a 0.51
- DP11 0.71
  - DP11a 0.59
- DP12 0.22
- DP13 0.51
  - DP13a 0.67
- DP14 0.69
  - DP14a 0.65

Decision points (DP) and corresponding proportion of disorientation averaged across all time segments belonging to the decision point and the participants. DPa,b indicate decision points in immediate proximity to a major decision point such as a crossing or landmark
